# Macrocytosis as an Early Pharmacodynamic Marker of Imatinib Efficacy in Chronic Myeloid Leukemia

**DOI:** 10.3390/jcm15020908

**Published:** 2026-01-22

**Authors:** Fatih Yaman, Ibrahim Ethem Pinar, Sevgi Isik, Filiz Yavasoglu, Eren Gunduz, Hava Uskudar Teke, Neslihan Andic

**Affiliations:** 1 Clinic of Hematology, Tokat Public Hospital, Tokat 60100, Turkey; 2 Department of Internal Medicine, Division of Hematology, Faculty of Medicine, Bursa Uludag University, Bursa 16059, Turkey; 3 Department of Medical Genetics, Faculty of Medicine, Eskisehir Osmangazi University, Eskisehir 26040, Turkey; sevgiozpolat@gmail.com; 4 Department of Internal Medicine, Division of Hematology, Faculty of Medicine, Eskisehir Osmangazi University, Eskisehir 26040, Turkey; drfilizyavasoglu@gmail.com (F.Y.); erengunduz26@gmail.com (E.G.); havaus@yahoo.com (H.U.T.); neslihandic@yahoo.com (N.A.)

**Keywords:** chronic myeloid leukemia, imatinib, mean corpuscular volume, macrocytosis, cytogenetic response, molecular response, treatment outcomes

## Abstract

**Background:** Macrocytosis commonly develops during imatinib therapy, but its relationship with cytogenetic and molecular outcomes in chronic myeloid leukemia (CML) remains unclear. We investigated whether increases in mean corpuscular volume (MCV) during imatinib treatment are associated with response depth and treatment persistence. **Methods:** In this retrospective study, we analyzed 101 adults with chronic-phase CML treated with a stable imatinib dose of 400 mg/day for at least 12 months. Patients with conditions that could confound MCV (hydroxyurea exposure, megaloblastic anemia, hypothyroidism, chronic liver disease, alcoholism) were excluded. Complete cytogenetic response (CCyR) and major molecular response (MMR) were assessed by conventional karyotyping and the BCR-ABL1 International Scale, respectively. Increased MCV was defined as MCV > 100 fL after six months of therapy, persisting thereafter. Associations between MCV dynamics, response, and switching to second-generation tyrosine kinase inhibitors were evaluated. **Results:** Twenty patients (20%) developed increased MCV. Overall, 86 patients (85%) achieved CCyR and 70 (69%) achieved MMR. All patients with increased MCV attained CCyR, compared with 66 of 81 (81%) without MCV elevation (*p* = 0.037), while MMR rates were 90% versus 64% (*p* = 0.030). During a median follow-up of 69 months, treatment modification was required in 1 of 20 (5%) patients with increased MCV versus 25 of 81 (31%) in the non-increased group (*p* = 0.018). **Conclusions:** MCV elevation during imatinib therapy is associated with deeper molecular response and reduced need for treatment modification. MCV dynamics may serve as an inexpensive pharmacodynamic marker to support risk assessment and guide monitoring in chronic-phase CML.

## 1. Introduction

Chronic myeloid leukemia (CML) is a clonal myeloproliferative neoplasm characterized by the BCR-ABL1 fusion tyrosine kinase generated by the Philadelphia (Ph) chromosome translocation t(9;22)(q34;q11), which drives uncontrolled proliferation of myeloid progenitor cells through constitutive activation of multiple downstream signaling pathways, including RAS/MAPK and JAK/STAT [1,2]. Before the advent of tyrosine kinase inhibitors (TKIs), CML was associated with limited therapeutic options and poor long-term survival. The introduction of imatinib, the first BCR-ABL1 TKI, fundamentally transformed the natural history of the disease, enabling most patients to achieve durable disease control and markedly improved survival compared with the pre-TKI era [3].

To refine therapeutic decision-making in the TKI era, several prognostic scoring systems—such as the Sokal and Hasford scores and, more recently, the EUTOS Long-Term Survival (ELTS) score—have been integrated into routine practice [4,5]. Unlike earlier models, ELTS focuses on CML-related mortality rather than cytogenetic response, providing a more contemporary framework for risk stratification. However, accumulating evidence suggests that readily obtainable hematologic parameters may also carry prognostic information. In particular, increased red blood cell distribution width (RDW) has been reported to be associated with inferior outcomes in chronic-phase CML patients receiving imatinib, raising the possibility that erythrocyte indices reflect aspects of disease biology or host response beyond conventional risk scores [6,7]. Although the underlying mechanisms remain incompletely understood, RDW has been hypothesized to mirror disordered erythropoiesis and systemic stress.

One hematologic alteration frequently observed during imatinib therapy is macrocytosis. In addition to inhibiting BCR-ABL1, imatinib suppresses c-KIT signaling, which plays a critical role in erythropoiesis at the burst-forming unit–erythroid (BFU-E) stage [8,9]. Impairment of c-KIT-mediated erythroid maturation is thought to contribute to increased mean corpuscular volume (MCV), and experimental data, largely derived from murine models, support this concept by demonstrating that loss of functional c-KIT leads to hematopoietic failure and severe macrocytic anemia [10].

Beyond hematologic malignancies, alterations in mean corpuscular volume have also been explored as potential prognostic or predictive markers in certain solid tumors, suggesting that MCV may reflect broader biological or treatment-related processes rather than disease-specific effects alone. In a cohort study of patients with esophageal cancer, MCV was found to be a predictive marker [11]. Macrocytosis has also been described with other TKIs that inhibit c-KIT, such as sunitinib and pazopanib, where it has been correlated with improved clinical outcomes, including prolonged progression-free survival in solid tumor populations [12,13]. Macrocytosis has been reported to be associated with adverse overall survival in patients with treatment-related acute myeloid leukemia [14]. Taken together, these observations suggest that TKI-induced macrocytosis may function as a broader pharmacodynamic marker reflecting the depth of kinase inhibition across different clinical settings.

In addition to disease- and treatment-related factors, host-dependent mechanisms are increasingly recognized as contributors to therapeutic response in leukemia. Recent studies have suggested a potential role of the gut microbiota in modulating treatment outcomes and immune responses across hematologic malignancies, underscoring the need to re-evaluate current concepts of response prediction beyond disease-intrinsic characteristics [15].

In the context of CML, increases in MCV during imatinib therapy have been associated with higher rates of complete cytogenetic response (CCyR) and a reduced likelihood of cytogenetic relapse [16]. In another study, the proportion of patients with increased MCV was significantly higher in the CCyR group than in the non-CCyR group. Moreover, patients with increased MCV were more likely to achieve and maintain major molecular response (MMR) [17]. These findings raise the possibility that dynamic changes in MCV may represent a simple, low-cost, and widely accessible surrogate biomarker of therapeutic efficacy. Nevertheless, data are limited, and the relationship between MCV elevation and distinct dimensions of treatment response—particularly molecular outcomes and long-term maintenance of remission—has not been fully characterized.

Accordingly, in this study, we aimed to evaluate the relationship between MCV elevation and treatment outcomes, including CCyR, MMR, and loss of cytogenetic remission, in patients with chronic-phase CML receiving imatinib.

## 2. Methods

### 2.1. Study Design and Setting

This single-center, retrospective study included adult patients (≥18 years) diagnosed with CML between 2010 and 2023 at Eskisehir Osmangazi University Faculty of Medicine. A total of 215 patients diagnosed with CML were initially screened for eligibility.

**Figure 1:**
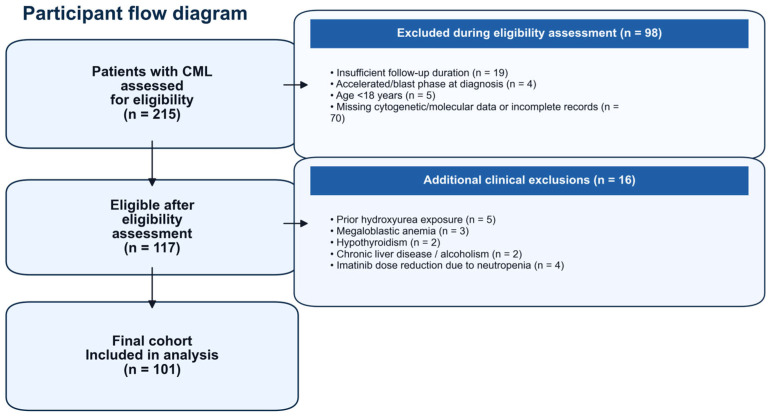
Flow diagram of patient selection and exclusion process. Flowchart showing patient screening, eligibility assessment, and stepwise exclusions for the retrospective chronic-phase chronic myeloid leukemia (CML) cohort treated with imatinib. Of 215 patients assessed for eligibility, 98 were excluded during eligibility assessment (insufficient follow-up, accelerated/blast phase at diagnosis, age < 18 years, or missing cytogenetic/molecular data/incomplete records). An additional 16 patients were excluded for clinical reasons that could confound mean corpuscular volume (MCV) interpretation (prior hydroxyurea exposure, megaloblastic anemia, hypothyroidism, chronic liver disease/alcoholism, or imatinib dose reduction due to neutropenia). The final analytic cohort comprised 101 patients receiving stable standard-dose imatinib (400 mg/day).

Patient selection was performed in a stepwise manner, as summarized in the flow diagram (Figure 1). During the eligibility assessment phase, patients were excluded due to insufficient follow-up duration (n = 19), accelerated or blast phase at diagnosis (n = 4), age < 18 years (n = 5) or missing genetic data and/or incomplete medical records (n = 70).

Following eligibility assessment, additional clinical exclusion criteria were applied, including prior exposure to hydroxyurea (n = 5), megaloblastic anemia (n = 3), hypothyroidism (n = 2), chronic liver disease or alcoholism (n = 2), and imatinib dose reduction due to neutropenia (n = 4). In all dose-reduced cases, imatinib was reduced to 300 mg/day due to neutropenia.

After application of these criteria, 101 patients who had received continuous imatinib therapy for at least one year were included in the final analysis. Only patients treated with a stable imatinib dose of 400 mg/day were eligible; those who required dose reductions at any time were excluded to ensure uniform treatment exposure. This restriction was applied to minimize confounding by heterogeneous dose intensity and to evaluate MCV dynamics under comparable drug exposure. Additional chromosomal abnormalities accompanying the Philadelphia chromosome at diagnosis were not considered exclusion criteria. The ELTS score was calculated at baseline for risk stratification. 

### 2.2. Cytogenetic and Molecular Assessments and Response Definitions

At diagnosis and during early follow-up, cytogenetic and molecular assessments were performed using bone marrow karyotyping, fluorescence in situ hybridization for the Philadelphia chromosome, and quantitative real-time polymerase chain reaction standardized to the International Scale (IS) for BCR-ABL1 transcript quantification. CCyR was defined as the absence of Ph-positive metaphases on conventional karyotyping, whereas MMR was defined as a BCR-ABL1 level ≤ 0.1% IS, in accordance with international criteria [18].

Fluorescence in situ hybridization (FISH) was performed according to the manufacturer’s instructions using a dual-color probe targeting the t(9;22)(q34;q11.2) translocation (ZytoVision GmbH, Bremerhaven, Germany). Hybridized slides were examined under a Nikon Eclipse 80i fluorescence microscope (Nikon Corporation, Tokyo, Japan). Digital images were captured and analyzed using CytoVision cytogenetic image analysis software, version 7.5 (Leica Biosystems, Newcastle upon Tyne, UK).

The p210 transcripts of the BCR::ABL1 fusion gene were analyzed by real-time reverse-transcription polymerase chain reaction (RT-PCR) using the manufacturer’s suggested protocol (geneMAP BCR-ABL1 p210 (Mbcr) Detection Kit [BCR210-RT48], GenMark Diagnostics, Irvine, CA, USA). RT-PCR amplification was performed on a LightCycler^®^ 480 real-time PCR system (Roche Diagnostics GmbH, Mannheim, Germany).

### 2.3. Definition of Study Groups

No external or healthy control group was included. All analyses were conducted within the same chronic-phase CML cohort to minimize inter-cohort variability. Patients were categorized a priori according to MCV dynamics during imatinib therapy into an increased MCV (macrocytosis) group and a reference (non-increased MCV) group. The laboratory reference range for MCV at our center was 80–95 fL. Increased MCV was defined as an MCV value > 100 fL at month 6 of continuous imatinib therapy that remained >100 fL on all subsequent measurements until last follow-up. The reference group comprised patients whose MCV was ≤100 fL at month 6 and remained ≤100 fL thereafter. This within-cohort grouping enabled assessment of associations between MCV dynamics and treatment outcomes while avoiding comparisons with external populations.

### 2.4. Data Collection

Demographic, clinical, laboratory, and genetic data were obtained retrospectively from institutional medical records. Baseline hematological parameters and molecular data at diagnosis were recorded when available. Patients with missing key genetic data required for analysis were excluded as described above.

### 2.5. Statistical Analysis

Statistical analyses were performed using IBM SPSS Statistics for Windows, version 24.0 (IBM Corp., Armonk, NY, USA) and Python (version 3.13.2; Python Software Foundation, Wilmington, DE, USA) with standard scientific libraries. The distribution of continuous variables was assessed using the Kolmogorov–Smirnov test. Comparisons between categorical variables, including cytogenetic and molecular response status, gender, ELTS risk category, and increased MCV status, were performed using the chi-square test. Parametric continuous variables were analyzed using Student’s *t*-test. Time to treatment modification was estimated using the Kaplan–Meier method, and survival differences between groups were compared with the log-rank test. All statistical tests were two-sided, and a *p*-value < 0.05 was considered statistically significant. Multivariable logistic regression models were fitted to evaluate independent associations between macrocytosis (increased MCV vs. normal) and binary outcomes at 12 months (CCyR and MMR), adjusting for age (per 1-year increase), sex (male vs. female), and ELTS risk (intermediate/high vs. low). When complete separation was present (i.e., there were no patients who did not achieve CCyR in the increased MCV group), Firth penalized logistic regression was applied. Results are reported as adjusted odds ratios (ORs) with 95% confidence intervals (CIs) and two-sided *p*-values.

## 3. Results

A total of 101 patients with chronic-phase CML receiving imatinib were included in the analysis. All patients achieved complete hematologic response within the first three months. The median age at diagnosis was 53 years (range, 18–87), and 56% were male. According to the ELTS score, 78% of patients were classified as low-risk. An increase in MCV during imatinib therapy was observed in 20 patients (20%), beginning after six months of treatment and remaining persistently elevated thereafter. Among the four patients excluded due to dose reduction to 300 mg/day for neutropenia, increased MCV was observed in one patient.

After 12 months of therapy, CCyR was achieved in 86 patients (85%). Patients in the increased MCV group were significantly older than those without MCV elevation (*p* = 0.045). All 20 patients with increased MCV achieved CCyR within the first year (*p* = 0.037). No significant associations were identified between CCyR and ELTS risk category or gender (*p* > 0.05). Multivariate analysis showed no significant predictive relationship between age, increased MCV, and CCyR attainment (*p* > 0.05). Clinical characteristics according to CCyR status and MCV group are summarized in Table 1 and Table 2.

Among the 86 patients who achieved CCyR, loss of cytogenetic response occurred in 11 individuals (12%) after a median follow-up of 69 months. Loss of CCyR was documented in 1 of the 20 patients (5%) in the increased MCV group and in 10 of the 66 patients (15%) in the normal MCV group, although this difference was not statistically significant (*p* > 0.05).

MMR at 12 months was achieved in 70 patients (69%). Eighteen of the 20 patients in the increased MCV group achieved MMR, and the proportion of patients achieving MMR was significantly higher in this group compared with those without MCV elevation (*p* = 0.03). MMR showed no significant association with age, ELTS risk category, or gender (*p* > 0.05). Patient characteristics according to MMR status are provided in Table 3.

**Figure 2:**
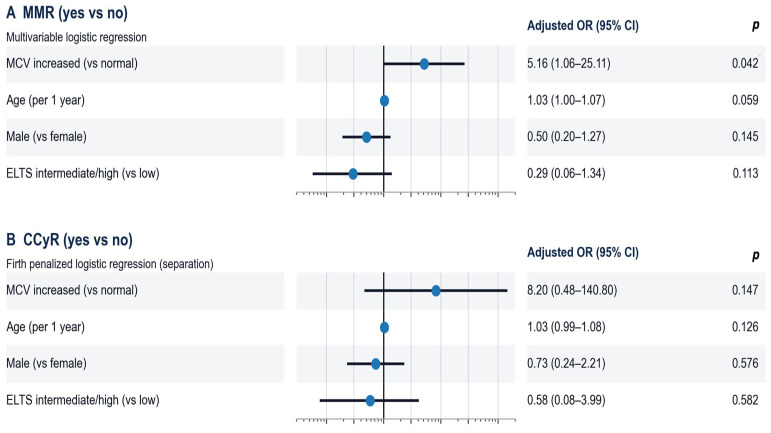
**Multivariable analyses of outcomes associated with macrocytosis (MCV increase) during imatinib therapy.** Forest plots show adjusted odds ratios (ORs) with 95% confidence intervals (CIs) on a log scale for (**A**) major molecular response (MMR) at 12 months, and (**B**) complete cytogenetic response (CCyR) at 12 months. All models included the same prespecified covariates: macrocytosis (MCV increased vs. normal), age (per 1-year increase), sex (male vs. female), and ELTS risk category (intermediate/high vs. low). Points indicate adjusted ORs and horizontal bars indicate 95% CIs; the vertical line denotes OR = 1. Two-sided p-values are shown in the right column. For the CCyR model, Firth penalized logistic regression was used due to complete separation (0-cell in the 2 × 2 table: there were no patients who did not achieve CCyR in the increased MCV group).

In multivariable logistic regression adjusted for age, sex, and ELTS risk category (intermediate/high vs. low), macrocytosis (increased MCV) remained independently associated with achievement of major molecular response at 12 months (OR 5.16, 95% CI 1.06–25.11; *p* = 0.042). For CCyR at 12 months, standard logistic regression was not appropriate due to complete separation (there were no patients who did not achieve CCyR in the increased MCV group); therefore, Firth penalized logistic regression was applied, showing a positive but non-significant association (OR 8.20, 95% CI 0.48–140.80; *p* = 0.147) (Figure 2).

Loss of MMR occurred in 10 of the 70 patients who had achieved MMR. This included 1 of 18 patients in the increased MCV group and 9 of 52 patients in the normal MCV group, with no significant relationship between increased MCV and loss of MMR (*p* > 0.05).

**Figure 3:**
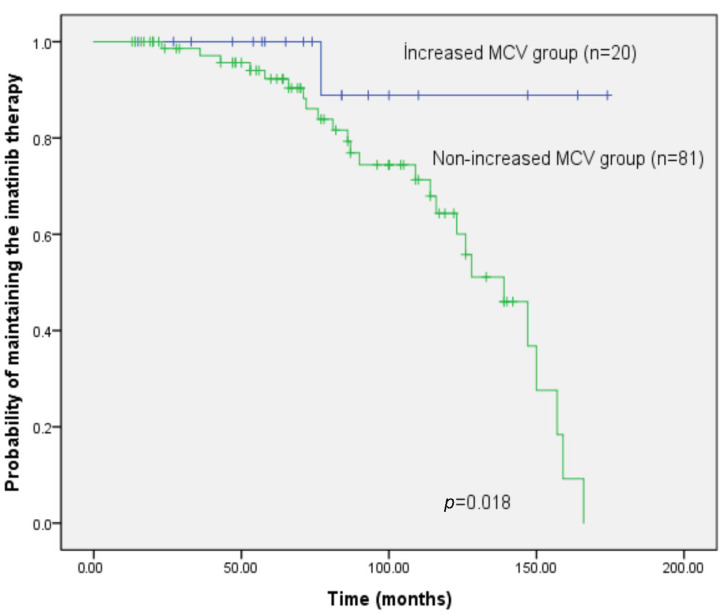
**Kaplan–Meier estimates of continued imatinib therapy according to MCV status.** Patients with increased MCV had a significantly higher probability of remaining on imatinib without treatment modification (*p* = 0.018).

When all patients were evaluated at 12 months of imatinib therapy, the proportion of individuals who either failed to achieve CCyR by the end of the first year or subsequently experienced loss of cytogenetic response during follow-up was significantly lower in the increased MCV group (*p* = 0.018). Imatinib was discontinued in only 1 of the 20 patients with increased MCV due to loss of CCyR, whereas 25 of the 81 patients in the normal MCV group required a switch to second-generation tyrosine kinase inhibitors because CCyR was not achieved at 12 months or was later lost during follow-up (Figure 3).

## 4. Discussion

The advent of TKIs has fundamentally altered the natural history of CML, yielding substantial improvements in hematologic, cytogenetic, and molecular outcomes. Imatinib, the first-generation TKI, remains a highly effective frontline agent and has demonstrated clear superiority over interferon-α-based therapies in randomized trials [19,20,21,22]. Achieving CCyR and MMR within the first year of therapy is widely recognized as one of the strongest predictors of long-term survival and durable treatment success.

Parallel to these therapeutic advances, increasing attention has been directed toward simple, accessible biomarkers that can predict response to imatinib. Proposed predictors include baseline prognostic scores such as the ELTS score, plasma imatinib concentrations, presenting disease burden, and erythrocyte indices [5,23]. Among these, RDW and MCV have emerged as promising hematologic markers due to their potential to reflect erythropoietic activity, marrow stress, or indirect measures of TKI exposure [6,7,24,25,26]. Elevated RDW has been associated with inferior outcomes in several studies and may reflect disordered erythropoiesis or abnormal proliferative signaling from leukemic stem cells, although its mechanistic underpinnings remain incompletely defined [24,25].

MCV elevation during imatinib therapy has gained prominence as a potential pharmacodynamic marker. Imatinib inhibits c-KIT in addition to BCR-ABL, thereby suppressing erythroid progenitor proliferation; higher imatinib plasma levels can reduce BFU-E colony formation and lead to macrocytosis [27]. The IRIS trial indirectly supports this mechanism, as anemia occurred more frequently in patients with greater imatinib exposure, suggesting a dose-dependent sensitivity of erythroid precursors to off-target kinase inhibition [22].

While macrocytosis during TKI therapy has been associated with faster achievement of CCyR, its relationship with cytogenetic and molecular responses remains inconsistent. In one retrospective study, treatment-emergent macrocytosis was associated with earlier complete hematologic response but showed no significant association with early molecular response or MMR, likely due to the low incidence of macrocytosis in the cohort [26].

In a landmark analysis, Song et al. demonstrated that increases in MCV after six months of therapy correlated with higher CCyR rates and fewer kinase domain mutations, raising the hypothesis that sustained macrocytosis may serve as a surrogate for adequate or prolonged drug exposure [16].

Consistent with our findings, previous reports have shown that MCV significantly increases during the first year of TKI treatment and that this increase correlates with favorable treatment outcomes. Patients with increased MCV were reported to achieve CCyR more frequently at 6 and were more likely to achieve CCyR at 6 and 12 months, with a trend toward improved molecular responses compared with those with decreased MCV [17].

In our cohort, increased MCV was not independently associated with CCyR at 12 months; however, it showed a significant association with achievement of MMR (*p* = 0.03). Patients with increased MCV achieved MMR at markedly higher rates (90%), consistent with prior findings suggesting that macrocytosis may parallel deeper molecular responses and more effective suppression of leukemic progenitors. These results support the concept that MCV elevation—an easily obtainable, cost-effective hematologic parameter—may function as an early indicator of adequate imatinib exposure and favorable molecular kinetics.

Long-term response dynamics further reinforce this interpretation. Treatment modification, defined as switching to a second-generation TKI, was substantially less frequent in the increased-MCV group, suggesting that patients who develop macrocytosis are less prone to cytogenetic relapse or treatment failure. Although we did not observe a statistically significant association between MCV elevation and subsequent loss of CCyR or MMR, this may reflect limited statistical power, particularly given the small size of the increased-MCV subgroup. Nevertheless, the markedly lower frequency of treatment change underscores the potential clinical utility of integrating MCV trends into routine monitoring, especially in contexts involving assessment of treatment adherence, dose adequacy, or evolving resistance patterns. Importantly, as patients requiring imatinib dose reduction were excluded, the findings of this study apply specifically to individuals receiving standard-dose imatinib and should not be extrapolated to dose-reduced populations.

Our findings show partial divergence from those of Song et al., who reported a stronger association between MCV elevation and cytogenetic outcomes [16]. Differences in sample size, treatment duration, patient demographics, monitoring frequency, or underlying mutation profiles may account for this variation. Despite these discrepancies, the overall trend in our cohort aligns with accumulating evidence that macrocytosis reflects adequate or sustained imatinib exposure and may serve as a pharmacodynamic signature of treatment effectiveness. Collectively, these findings highlight the value of MCV elevation as a practical, non-invasive, and potentially informative biomarker for early response assessment in patients receiving imatinib.

## 5. Limitations

This study has several limitations that should be acknowledged. First, the retrospective design precludes causal inference and may introduce selection bias. Second, the overall sample size—and particularly the relatively small number of patients with increased MCV—limited statistical power for less frequent outcomes (e.g., loss of CCyR/MMR) and contributed to imprecise estimates with wide confidence intervals. Accordingly, multivariable modeling was limited to a small set of clinically relevant covariates (age, sex, and ELTS risk), and for CCyR the presence of complete separation required Firth penalized logistic regression. Third, pharmacokinetic measurements and kinase domain mutation analyses were not uniformly available, restricting mechanistic interpretation of the association between MCV dynamics and response. Fourth, because patients requiring imatinib dose reduction were excluded to maintain uniform exposure, our findings apply specifically to patients treated with standard-dose imatinib (400 mg/day) and should not be extrapolated to reduced-dose regimens. Finally, as a single-center study, generalizability may be limited. Despite these limitations, the present analysis provides clinically relevant evidence supporting MCV dynamics as an accessible correlate of response during standard-dose imatinib therapy and warrants prospective validation in larger cohorts integrating pharmacokinetic and molecular data.

## 6. Conclusions

In conclusion, this study demonstrates that an increase in MCV during imatinib therapy is associated with superior molecular outcomes, particularly higher rates of major molecular response, and a substantially lower requirement for treatment modification. Although MCV elevation was not independently predictive of cytogenetic response at 12 months, patients exhibiting increased MCV values showed more durable treatment control and a reduced likelihood of switching to second-generation TKIs during long-term follow-up.

These findings suggest that MCV dynamics may reflect adequate or sustained imatinib exposure and effective suppression of leukemic activity, supporting its potential role as a simple, inexpensive, and readily available adjunct marker in routine clinical monitoring. Incorporating MCV trends into longitudinal assessment may aid clinicians in evaluating treatment adherence, dose sufficiency, and early response kinetics, particularly in settings where pharmacokinetic monitoring is not feasible.

Nevertheless, given the retrospective nature of the study and the absence of integrated pharmacokinetic and mutational data, MCV should be regarded as a supportive rather than definitive biomarker. Larger prospective studies with standardized molecular monitoring and pharmacodynamic correlations are required to validate the prognostic utility of MCV and to clarify its role within individualized treatment strategies, including considerations for treatment optimization and discontinuation in patients with sustained deep responses.

## Figures and Tables

**Table 1:** Baseline Characteristics Stratified by CCyR Status at 12 Months.

Characteristic	CCyR (n = 86)	Non-CCyR (n = 15)	*p*-Value
Age, median (range)	55 (18–87)	46 (18–78)	0.043
Gender			0.763
Male	48 (55%)	9 (60%)	
Female	38 (45%)	6 (40%)	
ELTS risk category			0.513
Low	66 (77%)	13 (87%)	
Intermediate/High	20 (23%)	2 (13%)	
MCV group			0.037
Increased MCV	20 (23%)	0 (0%)	
Non-increased MCV	66 (77%)	15 (100%)	

Abbreviations: CCyR, complete cytogenetic response; MCV, mean corpuscular volume.

**Table 2:** Clinical Characteristics According to MCV Group.

Characteristic	Increased MCV (n = 20)	Non-Increased MCV (n = 81)	*p*-Value
Age, median (range)	64 (25–86)	52 (18–87)	0.045
Gender			0.172
Male	14 (70%)	43 (53%)	
Female	6 (30%)	38 (47%)	
ELTS risk category			0.32
Low	14 (70%)	65 (80%)	
Intermediate / High	6 (30%)	16 (20%)	
CCyR at 12 months			0.037
Yes	20 (100%)	66 (81%)	
No	0 (0%)	15 (19%)	
MMR at 12 months			0.03
Yes	18 (90%)	52 (64%)	
No	2 (10%)	29 (36%)	

Abbreviations: MCV, mean corpuscular volume; CCyR, complete cytogenetic response; MMR, major molecular response.

**Table 3:** Patient Characteristics According to MMR Status.

Characteristic	MMR (n = 70)	Non-MMR (n = 31)	*p*-Value
Age, median (range)	55 (19–87)	51 (18–78)	0.116
Gender			0.276
Male	37 (52%)	20 (64%)	
Female	33 (48%)	11 (36%)	
ELTS risk category			0.897
Low	55 (78%)	24 (77%)	
Intermediate / High	15 (22%)	7 (23%)	
MCV group			0.03
Increased MCV	18 (25%)	2 (6%)	
Non-increased MCV	52 (75%)	29 (94%)	

Abbreviations: MMR, major molecular response; MCV, mean corpuscular volume.

## Data Availability

The datasets generated and/or analyzed during the current study are not publicly available due to ethical restrictions, but are available from the corresponding author upon reasonable request.
